# Efficacy of Platelet-Rich Plasma in Endoscopic Sinus Surgery for Chronic Sinusitis: A Systematic Review and Meta-Analysis

**DOI:** 10.7759/cureus.76568

**Published:** 2024-12-29

**Authors:** Jawad I Bukhari, Abdulrahman E Alghamdi, Shahad Mohammed Albeladi, Hala E Danish, Reem B Alqarni, Dakheelallah M Almutairi

**Affiliations:** 1 College of Medicine, King Saud Bin Abdulaziz University for Health Sciences, Jeddah, SAU; 2 Research Office, King Abdullah International Medical Research Center, Jeddah, SAU; 3 Department of Otolaryngology-Head and Neck Surgery, King Abdulaziz Medical City, Ministry of National Guard Health Affairs, Jeddah, SAU

**Keywords:** chronic sinusitis, endoscopic sinus surgery, meta-analysis, platelet-rich plasma, postoperative outcomes, sinusitis treatment, systematic review

## Abstract

Chronic rhinosinusitis (CR) is a persistent inflammation of the nasal mucosa and paranasal sinuses. Endoscopic sinus surgery (ESS) is a procedure that improves sinus drainage and ventilation. Despite advancements in ESS, additional corrective procedures post-ESS are often needed. Clinical trials have explored the efficacy of platelet-rich plasma (PRP) as adjunctive therapy after ESS. This study aims to provide evidence supporting the efficacy of PRP post-ESS for CS patients. Independent authors searched three electronic databases (Medline, EMBASE, and Cochrane Central Register of Controlled Trials (CENTRAL)) and assessed the methodological quality of included studies using the Cochrane risk of bias 2 tool (RoB 2). Only randomized controlled trials (RCTs) were included. We pooled standardized mean differences (SMDs) with corresponding 95% confidence intervals (CIs) using a random-effects model. Five RCTs met our inclusion criteria. Among 260 patients, 133 were allocated to PRP and 127 to the control group. PRP was associated with significantly lower postoperative nasal endoscopy scores (-1.74 (95% CI: -2.96, -0.52), P = 0.005) and Lund-Kennedy scores (-3.05 (95% CI: -4.97, -1.13), P = 0.002). PRP also significantly lowered Sinonasal Outcome Test-22 (SNOT-22) scores at one and three months follow-up (-8.25 (95% CI: -11.26, -5.24), P < 0.00001, and -2.75 (95% CI: -5.38, -0.12), P = 0.04, respectively). Subgroup analysis based on the location of administration showed borderline significance in the middle meatus group (-2.70 (95% CI: -5.35, -0.04), P = 0.05). This meta-analysis supports using PRP following ESS for CS patients. Despite promising results, further RCTs are needed to confirm long-term efficacy.

## Introduction and background

Chronic rhinosinusitis (CR) is a long-standing disease of the nasal mucosa and paranasal sinuses marked by inflammation [1]. Its diagnosis is established by at least two characteristic complaints of nasal discharge or postnasal drip, nasal congestion, facial pain or pressure, and a decreased sense of smell [2,3]. Confirmatory diagnosis is settled through computed tomography imaging or nasal endoscopy exhibiting signs of inflammation or purulent discharge in the background of more than 12 weeks. Initial medical management is to be followed with surgical treatment options if the former does not resolve inflammation [2].

The ensuing surgical modality is endoscopic sinus surgery (ESS) with the objectives of achieving sinus drainage and ventilation as well as mucociliary clearing. After surgery, patients are also expected to continue using intranasal corticosteroids (INCS) and saline rinses, which ESS plays a vital role in maintaining [1]. This field of management has been studied in abundance: studies show a notable improvement in symptoms such as nasal congestion and facial pain. Not only that but also ESS has been shown to amend Sinonasal Outcome Test-22 (SNOT-22) and quality of life (QOL) questionnaires [2]. Nevertheless, owing to the fact that there is a considerable chance of roughly 16% of requiring further corrective surgeries, it should be elucidated that ESS is not the ultimate, quick solution to chronic sinusitis [4].

Comprised of blood constituents such as platelets, bioactive agents, and growth factors, platelet-rich plasma (PRP) has been used in a multiplicity of medical cases across many specialties: this is due to their properties of regulating inflammation, healing wounds, and regenerating tissue [5-13]. Through this mechanism, it is hypothesized that PRP's utility following ESS can lessen the risk of incessant and recurring sinusitis and mitigate the development of recurrent nasal polyps [14].

The objective of this study is to systematically evaluate the clinical efficacy of platelet-rich plasma (PRP) as an adjunctive treatment following ESS in patients with chronic sinusitis. Given the variability in existing literature regarding the impact of PRP on surgical outcomes, a comprehensive meta-analysis of clinical trials is warranted. This meta-analysis aims to clarify the magnitude and direction of the efficacy of PRP, thus addressing the current gaps in understanding its clinical utility and benefits.

## Review

Methods

This systematic review followed the Preferred Reporting Items for Systematic Reviews and Meta-Analyses (PRISMA) and Cochrane review methods [15,16]. The protocol of this study was registered on PROSPERO (CRD42024581822) before conducting the initial search. Additionally, since the data is available online and the included studies had already obtained participants' consent, no further ethical approval or consent was required.

Eligibility Criteria

This systematic review encompassed all randomized controlled trials (RCTs) involving adults over 18 years old diagnosed with chronic sinusitis, with or without olfactory dysfunction, who underwent endoscopic sinus surgery and received platelet-rich plasma. The review considered studies reporting the efficacy of PRP, including insomnia duration, olfactory sensation score, and postoperative nasal endoscopy score. The review was restricted to English-language studies but placed no restrictions on the outcomes reported. Exclusion criteria included duplicates, case reports, conference abstracts, simulation studies, case series, review articles, molecular studies, and original reports other than randomized controlled trials. Furthermore, we excluded studies focused on interventions other than PRP and patients with systemic disorders, platelet function disorders, septal perforation, and unilateral sinus disease.

Search Strategy

We conducted a comprehensive systematic search of the Medline, EMBASE, and Cochrane Central Register of Controlled Trials (CENTRAL) without language or geographical restrictions from initiation until August 2024. The MeSH keywords used in the search engine included ((Platelet rich plasma OR Platelet-Rich Plasma OR Platelets rich plasma OR Platelets-Rich Plasma OR PRP OR Platelets enrich plasma OR Platelet-enrich plasma) AND (Functional endoscopic sinus surgery OR Endoscopic sinus surgery OR ESS OR FESS OR chronic rhinosinusitis OR rhinosinusitis OR chronic sinusitis OR sinusitis)). Furthermore, all references in the included articles were screened for relevance as well.

Study Selection and Data Extraction

The selection process was conducted by two independent reviewers who were blinded to the authors' names and journal titles. Initially, the reviewers independently screened the titles and abstracts of the identified articles to assess their relevance to the meta-analysis. Any discrepancies led to a full-text review of the articles. Subsequently, the full texts of the selected studies were evaluated for eligibility by the same two reviewers. Disagreements were discussed and resolved through consensus, with the senior authors consulted for final decisions on inclusion or exclusion. All studies were extracted following database searches and organized into an Excel spreadsheet (Microsoft Corp., Redmond, WA). The entire process, including methodological and statistical assessments, was carried out with careful attention to detail and collaboration among the reviewers.

Data Extraction and Study Outcomes

Data extraction was executed independently by two separate reviewers using a standardized data collection sheet on a Microsoft Excel sheet with any discrepancies resolved through discussion. The following elements were extracted from the study: study ID, setting, study duration, sample size, study arms, whether the population was self-controlled, and follow-up details. Furthermore, the characteristics of the populations were extracted, such as age, sex, administration methods, administration site, volume of blood collected, and techniques for PRP preparation. The outcomes for this meta-analysis included variables such as postoperative nasal endoscopy scores assessed using the Lund-Kennedy score, modified Lund-Kennedy score, and Meltzer score, Sinonasal Outcome Test-22 (SNOT-22), mean change of olfactory sensation score assessed using the modified Sniffin' Stick Test and Iran Smell Identification Test (I-SIT) score, and mean change of preoperative anosmia duration.

Risk of Bias Assessment

Two reviewers utilized the Revised Cochrane risk of bias 2 tool (RoB 2) to evaluate the risk of bias in the eligible RCTs independently [17]. Any disagreement was settled through discussion with a third author. This tool has various domains, and the judgments within each domain were carried forward for an overall RoB 2 judgment across five main domains. These domains are fixed, focusing on aspects of trial design, conduct, and reporting using a series of "signaling questions" to elicit information relevant to the risk of bias. This is then judged using an algorithm, and the judgments can be "low" (for all domains, the risk of bias is low), "some concerns" (for at least one of the domains, there is some concern), or "high" (for at least one domain has a high risk or some concerns for multiple domains).

Statistical Analysis

The data from the trials included in the analysis were evaluated using RevMan web-based software (The Cochrane Collaboration, London, UK). Effect sizes for the intended outcomes were combined using the standardized mean difference (SMD) or mean difference (MD) as appropriate with a random-effects model to calculate the overall effect size. A statistical significance threshold was set at a 95% confidence level (CI) and a P-value of less than 0.05. Statistical heterogeneity was assessed using the I² statistic and the P-values from the chi-square test. If the I² value exceeded 50%, a sensitivity analysis was conducted by excluding each study one at a time, and the highest change in the results was reported. Furthermore, subgroup analyses of postoperative nasal endoscopy scores based on different scoring systems, administration methods, and administration locations were conducted.

Results

Study Selection

**Figure 1:**
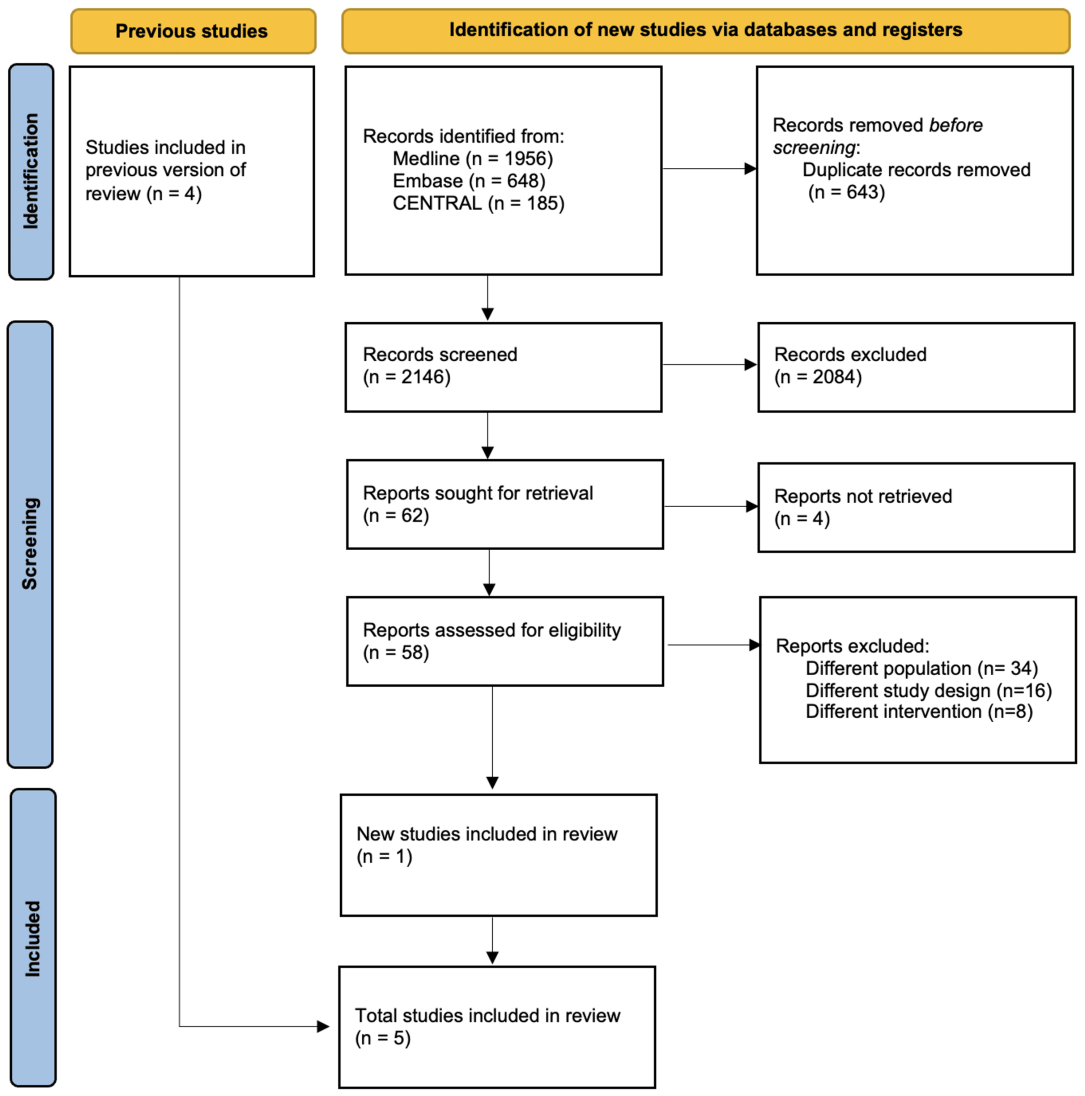
PRISMA flow diagram for the systematic search and trial selections PRISMA: Preferred Reporting Items for Systematic Reviews and Meta-Analyses, CENTRAL: Cochrane Central Register of Controlled Trials

A total of 2,789 records had been exported from the included databases. After removing the duplicates, the total number decreased to 2,146. These records have been investigated to decide whether to include or exclude them. A screening by abstract and title was done, in which 2,084 articles were excluded. Four out of the 62 articles were not retrieved. The remaining 58 articles underwent a full-text assessment in which 57 articles were excluded because they did not match our inclusion and exclusion criteria. Finally, five RCTs were included [18-22]. Further details are illustrated in Figure 1.

Study Characteristics

This systematic review includes a total of 260 participants. For gender distribution, some studies did not report the gender distribution number, which makes it difficult to determine the precise number. Overall, there were 77 males and 71 females, and that was calculated based on the reported numbers. In regard to study design, all included studies were RCTs. These studies have been conducted in Egypt, Turkey, Iran, and Greece. All studies focused on comparing the effectiveness of platelet-rich plasma (PRP) to no treatment or placebo. Moreover, they used a two-stage centrifugation technique for PRP preparation. Out of the five studies, two studies used irrigation as the method of PRP administration; the other three used direct injection. The effectiveness of the intervention was assessed during follow-up periods that ranged from one month to 12 months. Further details are shown in Table 1, which provides a summary of the included studies, and Table 2, which presents the baseline characteristics.

**Table 1:** Descriptive summary of the included studies PRP: platelet-rich plasma

Study ID	Study duration	Country	Sample size	Study arms		Population	Self-control	Follow- up
				Intervention	Control			
Dinaki et al. [18]	Between 2019 and 2023	Greece	N = 30	PRP	No PRP	Chronic rhinosinusitis with polyposis	No	12 months
Gökçe Kütük et al. [19]	Between 2019 and 2021	Turkey	N = 60	PRP	No PRP	Chronic paranasal sinus infection followed by olfactory dysfunction	No	3 months
Hassan et al. [20]	Between 2019 and 2020	Egypt	N = 40	PRP	Placebo	Chronic rhinosinusitis with or without polyps	Yes	1 month
Goljanian Tabrizi et al. [21]	Between 2017 and 2018	Iran	N = 48	PRP	Placebo	Chronic rhinosinusitis with polyposis and olfactory dysfunction	No	3 months
Mohebbi et al. [22]	Between 2016 and 2017	Iran	N = 21	PRP	No PRP	Chronic rhinosinusitis with polyposis	Yes	6 months

**Table 2:** Baseline characteristics of the included studies PRP: platelet-rich plasma, I-SIT: Iran Smell Identification Test

Study ID	Sample size		Age (years)		Sex (male/female)		Methods of PRP administration	Site of administration	Amount of blood collected	PRP preparation technique	Nasal endoscopy assessment tool	Olfactory sensation score tool
	PRP	Control	PRP	Control	PRP	Control						
Dinaki et al. [18]	15	15	45.47 ± 13.24	52.20 ± 7.64	Not reported	Not reported	Injection	Middle meatus and around the antrostomy	40 mL	Two-stage centrifugation	Modified Lund-Kennedy score	Greek version of the Sniffin' Stick Test
Gökçe Kütük et al. [19]	30	30	43.9 ± 7.38	42.63 ± 6.4	12/18	13/17	Injection	Olfactory region	Not reported	Two-stage centrifugation	Not reported	Modified Sniffin' Stick Test
Hassan et al. [20]	40	40	27 ± 11	27 ± 11	18/22	18/22	Irrigation	Middle meatus and ethmoidal cavity	20 mL	Two-stage centrifugation	Lund-Kennedy score	Not reported
Goljanian Tabrizi et al. [21]	27	21	37.15 ± 7.63	34.4 ± 6.95	19/8	15/6	Injection	Olfactory region	25 mL	Two-stage centrifugation	Not reported	I-SIT
Mohebbi et al. [22]	21	21	36.5 ± 7.91	36.5 ± 7.91	Not reported	Not reported	Irrigation	The surface where the polyps were removed	10 mL	Two-stage centrifugation	Meltzer scores	Not reported

Risk of Bias Assessment

**Figure 2:**
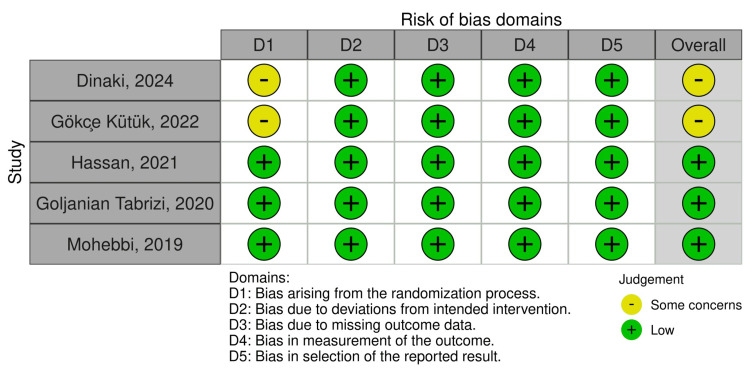
Risk of bias graph and summary of the included trials

The risk of bias was assessed by two reviewers simultaneously and separately. Two reviewers independently assessed the risk of bias across five eligible RCTs using the revised Cochrane risk of bias 2 tool (RoB 2) (Figure 2). The studies by Hassan et al. (2021) [20], Goljanian Tabrizi (2020) [21], and Mohebbi et al. (2019) [22] demonstrated a consistently low risk of bias across all assessed domains. In contrast, the studies by Dinaki et al. (2024) [18] and Gökçe Kütük et al. (2022) [19] showed some concerns in the "randomization process" domain, while the other four domains presented low risk of bias. Overall, the majority of the included studies can be viewed as having strong methodological quality, as evidenced by the low risk of bias assessments in all domains. However, the studies by Dinaki et al. (2024) [18] and Gökçe Kütük et al. (2022) [19] present some concerns related to the "randomization process" domain, suggesting potential limitations.

Postoperative Nasal Endoscopy Overall Score and at Different Time Points

**Figure 3:**
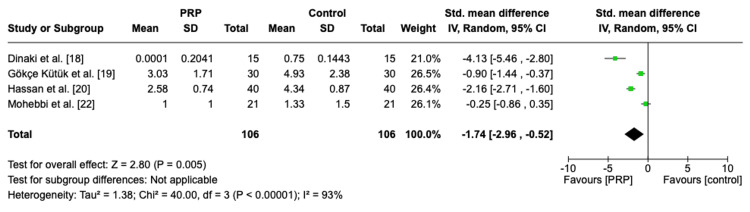
Forest plot of overall postoperative nasal endoscopy score PRP: platelet-rich plasma, SD: standard deviation, CI: confidence interval

**Figure 4:**
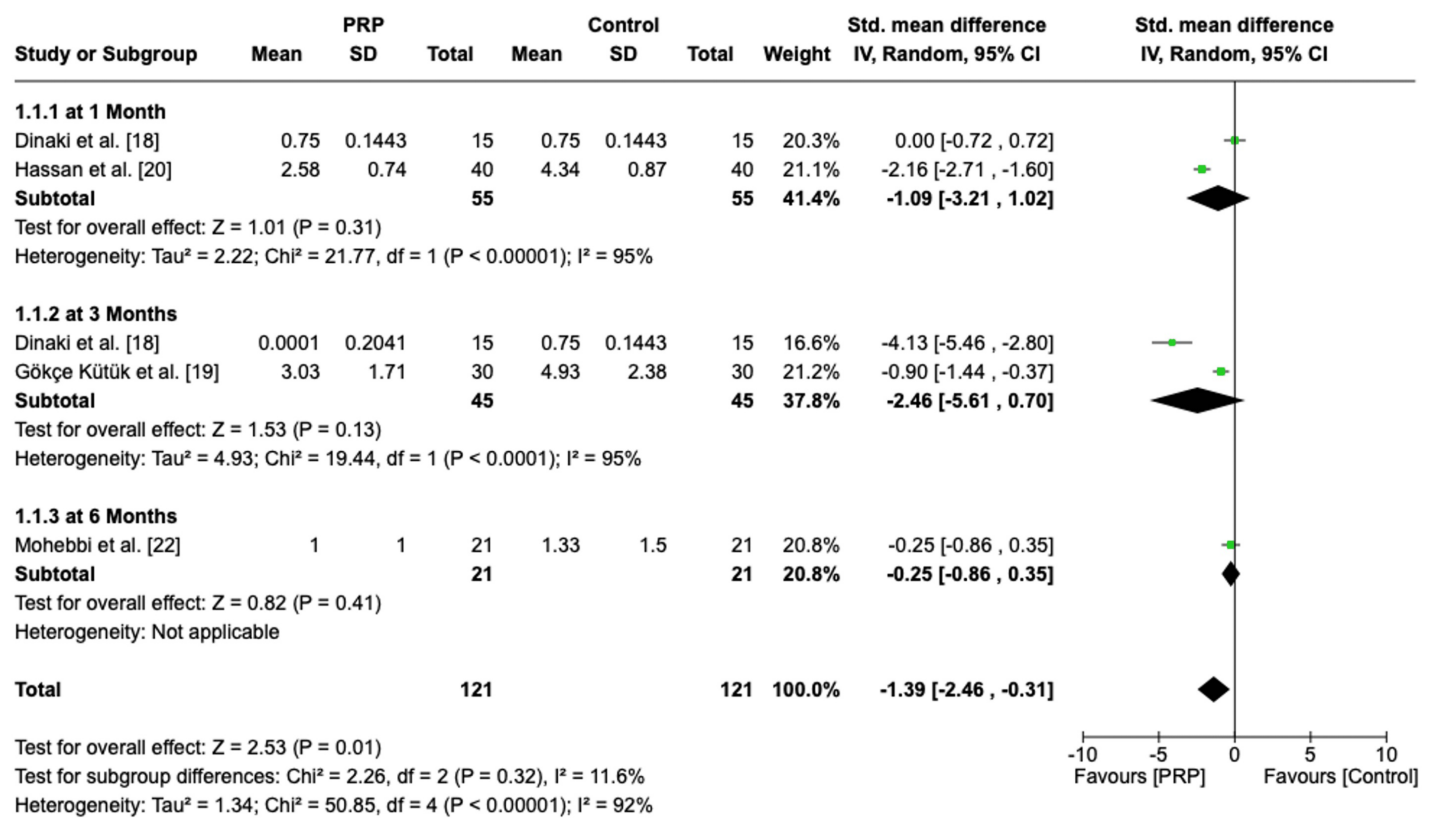
Forest plot of postoperative nasal endoscopy score at different time points PRP: platelet-rich plasma, SD: standard deviation, CI: confidence interval

A sum of 151 patients were included in our meta-analysis for assessing the efficacy of PRP. The overall pooled analysis demonstrated a significant effect size of -1.74 (95% CI: -2.96, -0.52, P = 0.005, I2 = 93%), implying that the score was in favor of the PRP group with heterogenous effects (Figure 3). The subgroup analysis at different time intervals showed no significant difference between the PRP group and the control group; however, it tended to favor PRP with heterogenous effects at one month (SMD = -1.09, 95% CI: -3.21, 1.02, P = 0.31, I2 = 95%), three months (SMD = -2.46, 95% CI: -5.61, 0.70, P = 0.13, I2 = 95%), and six months (SMD = -0.25, 95% CI: -0.86, 0.35, P = 0.41). Further details are shown on the forest plot in Figure 4.

Postoperative Nasal Endoscopy Score Based on Different Scoring Systems

**Figure 5:**
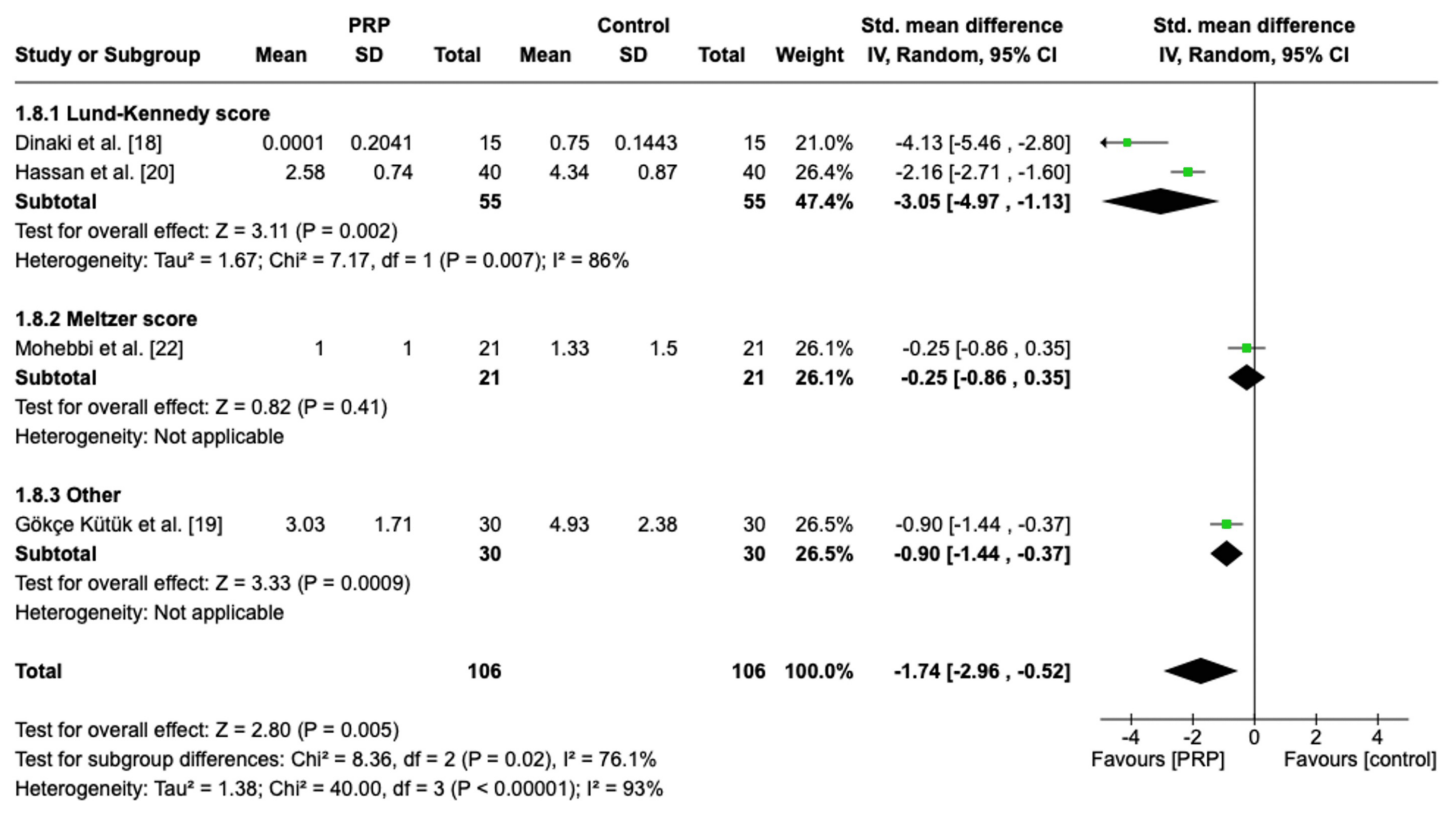
Subgroup analysis of postoperative nasal endoscopy score based on different scoring systems PRP: platelet-rich plasma, SD: standard deviation, CI: confidence interval

A subgroup analysis was conducted based on the scoring system, which included 151 patients. A significant overall effect favoring the PRP group was noticed in the Lund-Kennedy score group with heterogenous effects (SMD = -3.05, 95% CI: -4.97, -1.13, P = 0.002, I2 = 86%) and other scoring system group (SMD = -0.90, 95% CI: -1.44, -0.37, P = 0.0009). On the other hand, the Meltzer score showed no significant difference between the two groups (MD = -0.25, 95% CI: -0.86, 0.35, P = 0.41) (Figure 5).

Postoperative Nasal Endoscopy Score Based on the Administration Method

**Figure 6:**
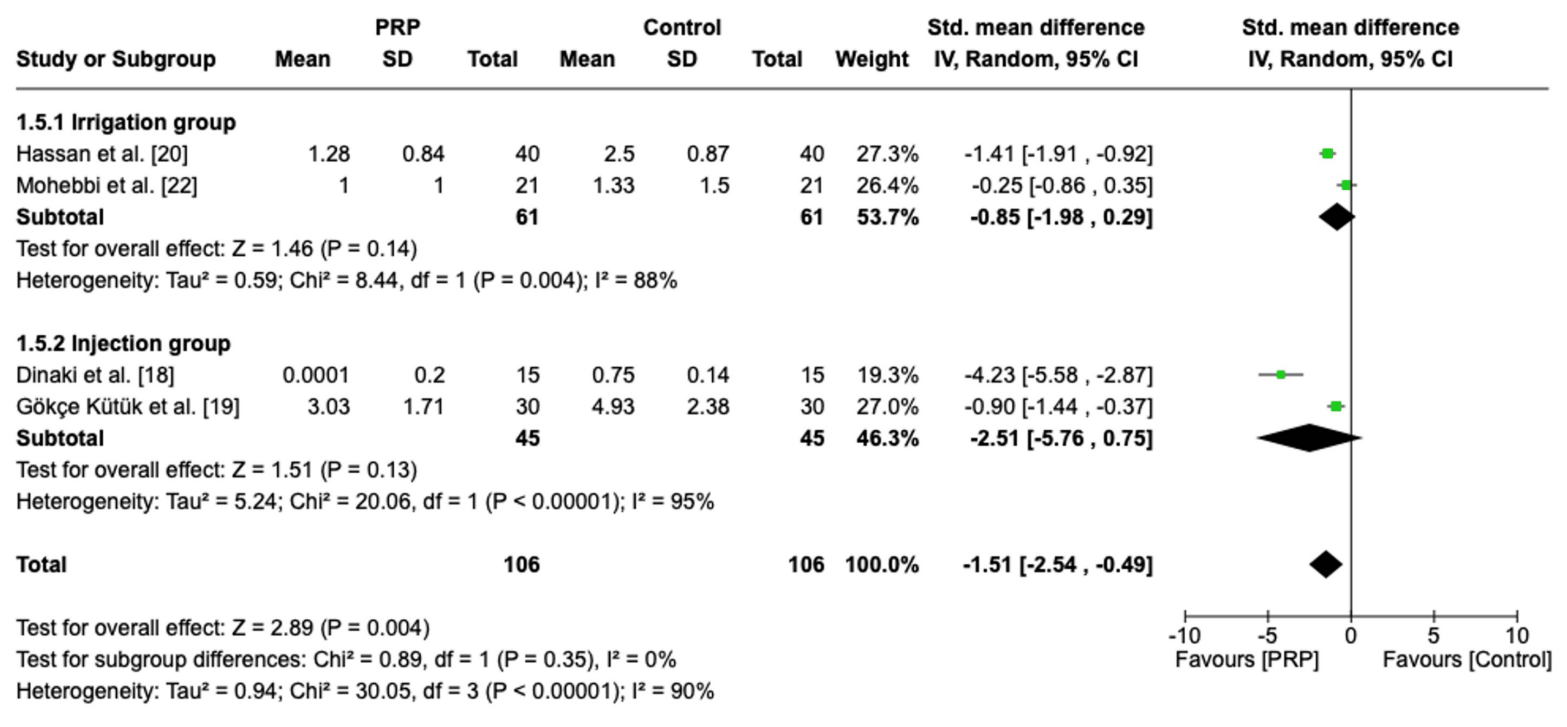
Subgroup analysis of postoperative nasal endoscopy score based on the administration method PRP: platelet-rich plasma, SD: standard deviation, CI: confidence interval

A subgroup analysis based on the administration method was performed. The pooled analysis of the irrigation group showed a standardized mean difference (SMD) of -0.85 (95% CI: -1.98, 0.29, P = 0.14, I2 = 88%), indicating no statistically significant difference between the PRP and control group with heterogenous effect. Similarly, the injection method yielded an SMD of -2.51 (95% CI: -5.76, 0.75, P = 0.13, I2 = 95%), which also showed no significant difference with high heterogeneity (Figure 6).

Postoperative Nasal Endoscopy Score Based on the Location of Administration

**Figure 7:**
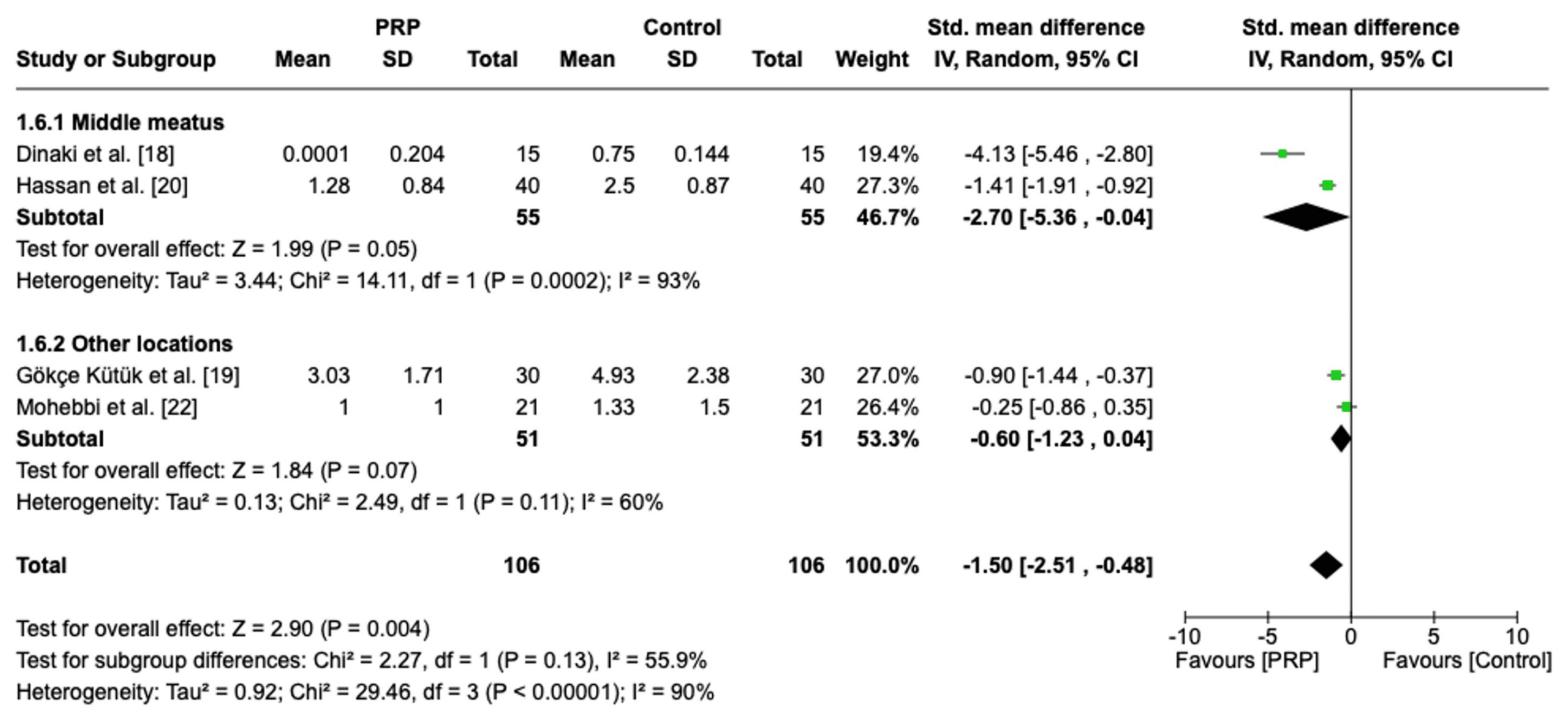
Subgroup analysis of postoperative nasal endoscopy score based on the location of administration PRP: platelet-rich plasma, SD: standard deviation, CI: confidence interval

A subgroup analysis based on the location of administration was performed. The pooled analysis of PRP administered in the middle meatus group demonstrated a difference with borderline significance and heterogenous effects (SMD = -2.70, 95% CI: -5.35, -0.04, P = 0.05, I2 = 93%). Regarding the pooled analysis of other locations group, PRP had a positive but non-significant effect with moderate heterogeneity (SMD = -0.60, 95% CI: -1.23, 0.04, P = 0.07, I2 = 60%) (Figure 7).

SNOT-22 Score at Different Time Points

**Figure 8:**
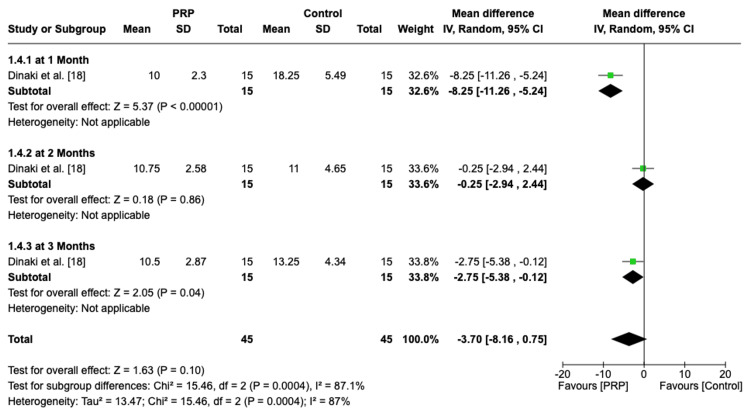
Forest plot of SNOT-22 score at different time points PRP: platelet-rich plasma, SD: standard deviation, CI: confidence interval, SNOT-22: Sinonasal Outcome Test-22

The analysis of a single RCT revealed that PRP significantly improves SNOT-22 scores at one month (MD = -8.25, 95% CI: -11.26, -5.24, P < 0.00001). Similarly, at three months, PRP also showed a statistically significant improvement (MD = -2.75, 95% CI: -5.38, -0.12, P = 0.04). However, at two months, no significant difference is observed (MD = -0.25, 95% CI: -2.94, 2.44, P = 0.86) (Figure 8).

Mean Change of Olfactory Sensation Score and Sensitivity Analysis

**Figure 9:**
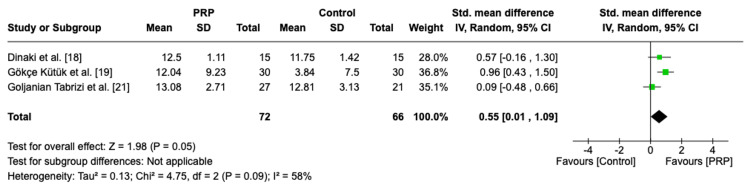
Forest plot of mean change of olfactory sensation score PRP: platelet-rich plasma, SD: standard deviation, CI: confidence interval

**Figure 10:**
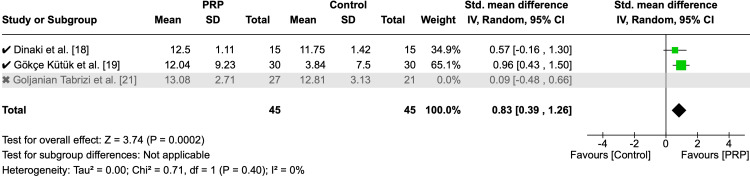
Sensitivity analysis of mean change of olfactory sensation score PRP: platelet-rich plasma, SD: standard deviation, CI: confidence interval

Three RCTs with a total number of 138 patients assessed the olfactory sensation score. The pooled analysis demonstrated a borderline significance with moderate heterogenous effect favoring PRP over control (MD = 0.55, 95% CI: 0.01, 1.09, P = 0.05, I² = 58%) (Figure 9). In sensitivity analysis, after excluding one study, the analysis revealed a highly significant effect in favor of PRP with a homogenous effect (MD = 0.83, 95% CI: 0.39, 1.26, P = 0.0002, I² = 0%) (Figure 10).

Mean Change of Preoperative Anosmia Duration

**Figure 11:**
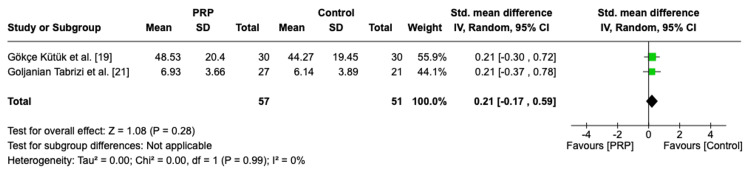
Forest plot of mean change of preoperative insomnia duration PRP: platelet-rich plasma, SD: standard deviation, CI: confidence interval

The pooled analysis of preoperative anosmia duration across two RCTs with a total of 108 patients showed no statistically significant difference between the PRP and control groups with a homogenous effect (SMD = 0.21, 95% CI: -0.17, 0.59, P = 0.28, I2 = 0%) (Figure 11).

Discussion

This systematic review and meta-analysis incorporated five randomized controlled trials with a total of 199 subjects to evaluate the efficacy of platelet-rich plasma in improving postoperative outcomes after endoscopic sinus surgery. The included studies in this review utilized a two-stage centrifugation technique to prepare PRP and compared its effects to control treatments. PRP significantly enhanced postoperative nasal endoscopy scores; subgroup analyses by time point, administration method, and location yielded mixed results. Although some subgroups favored PRP, these differences did not always reach statistical significance. In addition, SNOT-22 scores demonstrated significant improvement with PRP at one and three months post-surgery. Moreover, olfactory function showed borderline significance favoring PRP, which became highly significant following sensitivity analysis. However, PRP did not significantly impact the duration of preoperative anosmia.

The discrepancies observed in the various analyses may be attributed to variations in the demographics or characteristics of the patients involved, how the treatments were administered, and the lengths of the follow-up periods. The studies included patients with varying conditions, such as chronic sinusitis with or without olfactory disorders and sinonasal polyposis, leading to differences in baseline characteristics. PRP was administered using various methods, including submucosal injections and nasal packing, or as adjunctive therapy. There were differences in the concentration of PRP and the specific delivery location. Outcome measures also differed, with some studies focusing on wound healing and others on olfactory function, and varying follow-up periods, ranging from weeks to months, further contributing to variability. Additionally, geographical factors such as cultural and healthcare system variations due to different countries also introduced further inconsistencies and variabilities when pooling the results together in the meta-analysis.

PRP's healing effects come from its high levels of growth factors and cytokines, which are essential for tissue repair and healing. These growth factors include platelet-derived growth factor (PDGF), transforming growth factor-beta (TGF-β), and vascular endothelial growth factor (VEGF). In the process of wound healing, they play a crucial role in enhancing the recruitment, proliferation, and differentiation of cells such as fibroblasts and endothelial cells [23]. This speeds up tissue repair and reduces inflammation after surgery. Compared to traditional healing methods, PRP offers a concentrated and targeted approach, which can enhance the speed and quality of recovery [24]. Also, PRP is taken from the patient's own body, reducing the risk of immune reactions and infections and making it a safer option than synthetic growth factors or grafts. Additionally, the presence of leukocytes in PRP adds antimicrobial properties, creating a healthy healing environment and decreasing the chance of postoperative infections and complications [25,26].

PRP has demonstrated potential in improving postoperative outcomes, particularly in enhancing nasal endoscopy scores, olfactory function, and quality of life as measured by SNOT-22 scores. Clinically, PRP's regenerative properties are most likely the cause of these improvements, as it is rich in growth factors that promote tissue repair, reduce inflammation, and facilitate the healing process within the nasal mucosa. This aligns with our findings, which show that PRP significantly improved nasal endoscopy scores, especially when using the Lund-Kennedy scoring system. However, that significance was due to an elevated heterogeneity, which indicates the variability in patient responses to the intervention. Thus, the benefits of PRP may vary depending on individual factors such as the specific technique used, the characteristics of patients, and the duration of treatment. Regarding olfactory function, PRP's potential to regenerate olfactory receptor neurons could explain the borderline significant improvement in olfactory sensation scores observed in our study. This is further supported by the sensitivity analysis, which revealed a highly significant effect favoring PRP with homogenous results. This finding was consistent with the findings of Abo El Naga et al., who also reported a notable improvement in olfactory function [27]. However, this contrasts with Albazee et al., who found only modest improvements [14], highlighting the ongoing debate about the extent of PRP's effectiveness in treating olfactory dysfunction.

The significant improvements in SNOT-22 scores at one and three months post-treatment indicate that PRP may effectively alleviate the symptoms of chronic rhinosinusitis, thus improving patients' overall quality of life. However, the lack of significant improvement at the two months suggests that the benefits of PRP might manifest differently across patients. This phenomenon is best explained by the fact that differences in individual healing rates or the severity of underlying conditions will affect response to treatment. On the other hand, the absence of a significant effect of PRP on the duration of preoperative anosmia, as observed in both our study and the findings of Albazee et al. [14], suggests that while PRP may help improve olfactory function, it may be less effective in altering the course of long-standing olfactory dysfunction when compared with previous studies. Our findings support the growing evidence that indicates PRP's role as a beneficial adjunctive therapy in nasal surgery, particularly in improving postoperative outcomes and enhancing recovery. However, the variability in responses and the need for standardized protocols emphasize the importance of further research that aims to optimize PRP application, determine the most effective administration techniques, and identify patient subgroups most likely to benefit from this treatment.

This meta-analysis had several limitations. First, this meta-analysis was conducted on an aggregate level of data rather than individual patient data, introducing potential constraints on the precision of our findings. Second, heterogeneity among the pooled RCTs presents a substantial challenge in formulating definitive conclusions regarding the efficacy of PRP. Potential sources of heterogeneity encompass variations in patient selection criteria, scoring systems, treatment durations, and geographical locations. Third, the limited number of the included RCTs and a limited number of participants in each study may affect our conclusion's robustness. Fourth, the variability in PRP administration and outcome measurement highlights the need for standardized protocols in future research to ensure more consistent and reliable results. Finally, the risk of bias assessment revealed some concerns, particularly in the randomization process in two of the included studies, which could introduce some bias when pooling the results. Thus, interpretation of the results should be with caution.

## Conclusions

Our meta-analysis supports using platelet-rich plasma following endoscopic sinus surgery for patients with chronic rhinosinusitis. We observed improvements in nasal endoscopy scores, olfactory function, and quality of life. Despite promising results, variability across studies suggests the need for further research to standardize PRP protocols and confirm its long-term efficacy in this setting.
